# The metabolic equivalents of one-mile walking by older adults; implications for health promotion

**DOI:** 10.15171/hpp.2017.38

**Published:** 2017-09-26

**Authors:** Mandy Lucinda Gault, Mark Elisabeth Theodorus Willems

**Affiliations:** Department of Sport and Exercise Sciences, University of Chichester, College Lane, Chichester, UK

**Keywords:** One-mile walk, Metabolic equivalent, Health promotion, Aging

## Abstract

**Background:** Instructions for older adults regarding the intensity of walking may not elicit an intensity to infer health gains. We recorded the metabolic equivalents (METs) during a 1-mile walk using constant and predicted values of resting MET in older adults to establish walking guidelines for health promotion and participation.

**Methods:** In a cross-sectional design study, participants (15 men, 10 women) walked 1-mile overground, in a wooden floored gymnasium, wearing the Cosmed K4b^2^ for measurement of energy expenditure. Constant or predicted values for resting MET were used to calculate the number of 1-mile walks to meet 450-750 MET∙min∙wk^-1^.

**Results:** Participants had MET values higher than 3 for both methods, with 29% and 64% of the participants higher than 6 for a constant and predicted MET value, respectively. The METs of the1-mile walk were (mean ± SD) 6 ± 1 and 7 ± 1 METs using constant and predicted resting MET,and similar for men (constant: 6 ± 1 METs; predicted: 7 ± 1 METs) and women (constant: 5±1METs; predicted: 6 ± 1 METs) (P > 0.05).

**Conclusion:** Older adults that are instructed to walk 1-mile at a fast and constant pace meet the minimum required intensity for physical activity, and public health guidelines. Health professionals, that administer exercise, could encourage older adults to accumulate between six and nine 1-mile walks per week for health gains.

## Introduction

Health promotion guidelines suggest that healthy adults benefit from at least 150-minutes of moderate-intensity activities per week, achieved by bouts of at least 10 minutes, to reduce risk of chronic diseases and maintain quality of life.^1,2^ Walking is a popular form of physical activity and suitable for all population groups.^3^ However, instructions regarding the intensity may not elicit an intensity required to infer health gains in older adults.^4^ The metabolic equivalent (MET), a method of estimating energy expenditure during physical activity is used to prescribe exercise.^5^ The 1-MET represents energy expenditure while sitting quietly,^6^ with the assumption that it is equal to 3.5 ml∙kg^-1^∙min^-1^ as reported by Jetté et al,^7^ and defined here as the M1 method. However, Byrne et al^8^ observed that resting V̇O_2_ was 35% lower than the commonly accepted 1-MET value of 3.5 mL∙kg^-1^∙min^-1^. Therefore, 1-MET needs to be individualised with consideration of body mass index, age and gender,^8^ a measurement applicable in the community environment, defined here as the M2 method. Hall et al^9^ demonstrated that older adults have 1-MET values 31% lower than 3.5 mL∙kg^-1^∙min^-1^. Therefore, more research is required to better understand the frequency, intensity and duration of exercise prescription for older adults, and establish more specific public health guidelines for exercise professionals to promote.

The energy cost of physical activities is expressed as multiples of 1-MET, and considered light (i.e. <3-METs), moderate (i.e. 3-6-METs) or vigorous (i.e. >6-METs) intensity.^7^ Physical activity guidelines recommend that adults should accumulate 450-750 MET∙min∙wk^-1^.^6^ However, the general public may not understand what this means for their exercise practice, making it difficult to identify if they have completed enough exercise. Therefore, M1 and M2 are simple methods that can be used to simplify public health recommendations to achieve 450-750 MET∙min∙wk^-1^ with 1-mile walks. According to Fitzsimons et al,^4^ instructions to walk at a fast pace for 150 meters achieved an intensity of 4.3 METs (moderate intensity), sufficient to infer health gains. It is not known whether instructions to walk 1-mile as fast as possible will allow older adults to achieve a sufficient intensity and allow to complete multiple 1-mile walks to meet physical activity recommendations, which could be a simple and easy health promotion method for older adults to follow and monitor.

Therefore, the aim of the present study was to examine if instructions to walk fast during the 1-mile walk would result in achieving the intensity guidelines that incur health benefits for elderly men and women. Also, how many 1-mile walks would be required per week to meet physical activity of 450-750 MET∙min∙wk^-1^ in elderly men and women based on the M1 and M2 method for 1-MET.

## Material and Methods

### Participants and settings

Twenty-five healthy older adults (mean age: 67 [SD 4] years) were recruited within the local community for this cross-sectional study. Participants completed a health history questionnaire and were in good health with four on medications to lower cholesterol. Ethical approval was obtained from the University Research Ethics Committee (Code: REC260697), and written informed consent was obtained after an explanation of the procedures and risks. Procedures followed were in accordance with the Declaration of Helsinki of 1975, as revised in 2008.

### Procedures

Height (Holtain Ltd, Crymych, U.K.) and body mass (Seca Model 880, Seca Ltd., Birmingham, UK) were measured to the nearest cm and 0.1 kg, respectively, with participants lightly clothed and shoeless. Body mass index (BMI) was calculated and body fat percentage measured with bioelectrical impedance analysis (BF%; BC418 MA, Tanita, U.K.). Participants completed two indoor timed 1-mile walks individually, one familiarisation^10^ and one wearing the Cosmed K4b^2^ portable metabolic system, with a maximum of 1-week between. The timed 1-mile walk was used to predict maximal oxygen uptake using end heart rate (Polar FS1, Polar U.K.), age, and gender.^11^ Participants completed 28½ laps around a wooden floored gymnasium (19x9 m) with recording of step frequency at minutes 1, 5 and 10. Instructions were to complete the 1-mile walk at a fast constant pace. No encouragement was provided but participants were informed how many laps remained.^10^

### Energy expenditure

Energy expenditure and heart rate were measured using the Cosmed K4b^2^ portable metabolic system throughout the duration of the 1-mile walk (Cosmed K4b^2^, Cosmed, Rome, Italy). Wearing the Cosmed K4b^2^ portable metabolic system has no effect on the performance of older adults completing the one-mile walk.^12^ Calibration on each testing day was with standard gases of known concentrations (oxygen, O_2_: 15.6%, carbon dioxide, CO_2_: 5.66%; Linde Gas, UK). Initially, a room air measurement calibration was conducted (O_2_: 20.93%, CO_2_: 0.03%) and respiratory volume was calibrated using a 3 L volume syringe. A delay calibration was used in order to match the changes in fractions of expired oxygen (i.e. F_E_O_2_) and fractions of expired carbon dioxide (i.e. F_E_CO_2_). Method 1 (i.e. M1) used the one MET of Jetté et al^7^, i.e. 3.5 mL∙kg^-1^∙min^-1^. Method 2 was that of Byrne et al^8^ with 1-MET calculated by 3.6145 – (0.0367 x BMI) – (0.0038 x age) + (0.1790 x gender), where for gender, female = 1 and male = 2. Heart rate and oxygen uptake values (V̇O_2_) reflect averages taken over the duration of the 1-mile walk. 1-MET values from M1 and M2 were used to calculate maximal METs from 1-mile walk predicted V̇O_2_max, and METs during the 1-mile walk from mean V̇O_2_ as recorded by the Cosmed K4b^2^.

### Statistical analyses

Data were presented as mean ± SD for all parameters. V̇O_2_max was estimated using a generalized L∙min^-1^ equation by Kline et al^11^: V̇O_2max_ = 6.9652 + (0.0091 x WT) – (0.0257 x AGE) + (0.5955 x SEX) – (0.2240 x T) – (0.0115 x HR), where WT = body weight in pounds, AGE = years, T = 1-mile time in minutes to 100ths of a minute, HR = heart rate determined at the completion of the walk (beats∙ min^-1^), SEX = female 0, male 1. Maximum heart rate was predicted using the equation HRmax = 208-0.7 x AGE.^13^ Statistical Package for the Social Sciences version 16.0 (SPSS Inc., Chicago, IL, USA) for Windows was used for statistical analyses, including normality checks on all data sets, proceeded by independent samples *t* test’s to determine differences in METs between M1 and M2, and between men and women during the 1-mile walk and predicted maximum oxygen uptake and Pearson Moment correlations (step frequency, time to complete 1-mile and walking speed during 1-mile). Significance level was set at 0.05. The figures were drawn by Graph Pad Software (https://www.graphpad.com).

## Results

Mean age (men: 66 [SD 4], women: 67 [SD 4] years), BMI (men: 26.6 [SD 3.9], women: 26.0 [SD 3.0] kg·m^-2^) and body fat (men: 28.6 [SD 7.0], women: 33.7 [SD 6.2] %) were similar for men and women. Men were taller (men: 175 [SD 3], women: 162 [SD 7] cm, *P *< 0.05) and had higher body weight (men: 81.7 [SD 11.0], women: 68.7 [SD 10.8] kg, *P *< 0.05). Five men and 4 women were classified as normal weight according to BMI, 7 men and 4 women overweight and 3 men, 2 women obese. According to BF%, 4 men were average (≤24%) and 6 women (≤31%), with 11 men above average (≥25%) and 4 women (≥32%).

### One-mile walk

**Figure 1:**
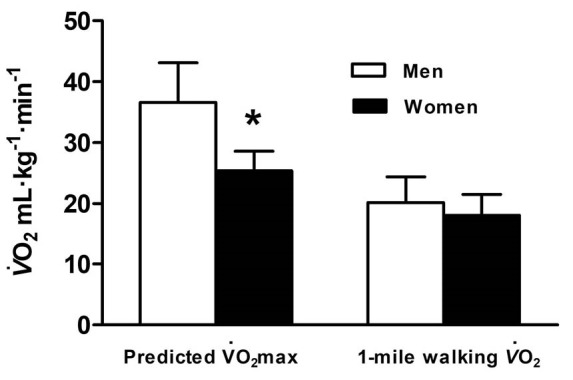
Predicted maximal oxygen uptake (V̇O_2_max) and walking oxygen uptake (V̇O_2_) of men and women during the 1-mile walk test. Data are presented as mean ± SD. * Significant difference between men and women, *P*<0.05.

The 1-mile walk was completed in 14 minutes and 48 seconds (i.e. 888 [SD 90] seconds, range: 12 minutes 10 seconds [730 seconds] – 19 minutes 15 seconds [1155 seconds] with the walking speed 1.86 [SD 0.24] m·s^-1^). The mean step frequency was 130 (SD 9) steps·min^-1^ (range: 113-146 steps·min^-1^). Men had a lower mean step frequency (127 [SD 9] steps·min^-1^) than women (135 [SD 6] steps·min^-1^) during the walk (t_(23)_=2.25, *P *= 0.03). Pearson correlations show a moderate significant negative correlation between step frequency and time to complete the 1-mile walk as fast as possible (*r* = -0.41, *P *= 0.04), and a moderate positive correlation between step frequency and 1-mile walking speed (*r* = 0.46, *P *= 0.02). Predicted max was higher for men than women (t_(23)_=-5.04, *P *< 0.001; Figure 1). Heart rate during the 1-mile walk was 116 (SD 16) beats∙min^-1^ for men, significantly lower than women (134 [SD 16] beats∙min^-1^; t_(23)_ = 3.09, *P *= 0.005). Both men and women had a similar age-predicted maximum heart rate (161 [SD 3] beats∙min^-1^). Indicative that women were exercising at a higher percentage of their age-predicted HRmax (83 [SD 7] %) than men (72% [SD 10]; t_(23)_ = 3.06, *P *= 0.006).

### Metabolic equivalent

**Figure 2:**
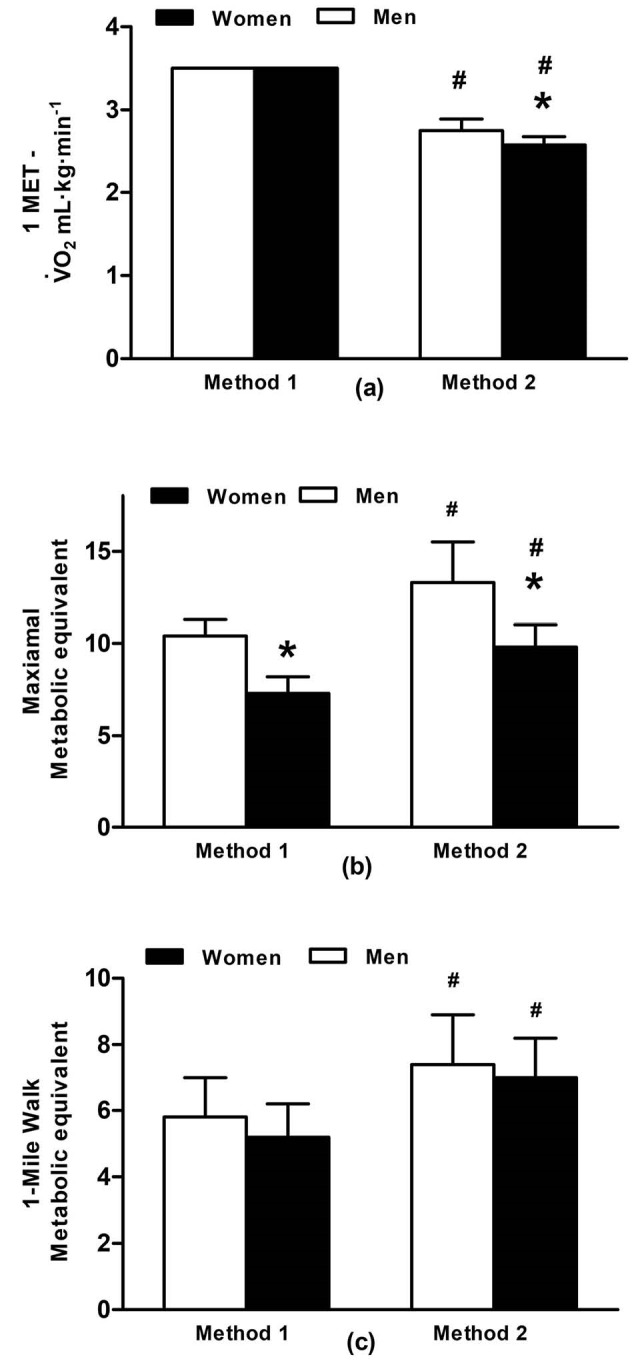
(a) Resting metabolic equivalent (1-MET); (b) Maximal metabolic equivalent and; (c) Metabolic equivalent of the 1-mile walk for men and women using the Jetté et al^7^ (M1) and Byrne et al^8^ methods (M2). Data are presented as mean ± SD, *P *<0.05. * Significant difference between men and women; # Significant difference between M1 and M2.

1-MET, maximal METs and 1-mile walk METs for M1 and M2 are presented in Figure 2. Men had significantly higher 1-MET than women (t_(23)_=-3.20, *P *= 0.004) with M2 being 31% (SD 7) lower than M1 (Figure 2a) (t_(24)_=-28.00, *P *< 0.001). M2 provided a range of 2.47-2.97 mL∙kg^-1^∙min^-1^ for men and 2.44-2.71 mL∙kg^-1^∙min^-1^ for women. Men had greater maximal METs than women for both M1 (t_(23)_=-4.88, *P *< 0.001) and M2 (t_(23) _= -4.50, *P *< 0.001) (Figure 2b). In addition, M2 produced higher maximal METs for both men and women than M1 (t_(24)_= 19.80, *P *< 0.001) (Figure 2b).

**Figure 3:**
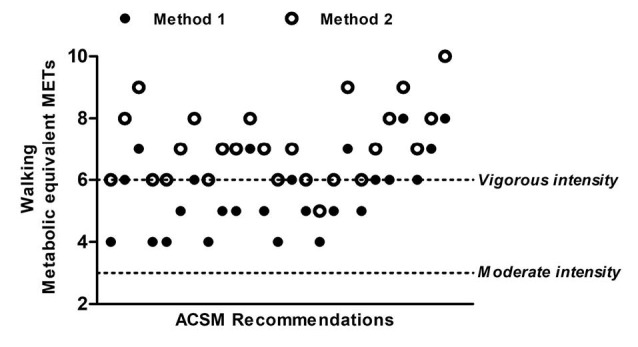
Individual metabolic equivalence during the 1-mile walk using Jetté et al^7^ constant (M1) and Byrne et al^8^ predicted 1-MET values (M2) and their relationship to the American College of Sports Medicine recommendations for moderate and vigorous intensity exercise.

During the 1-mile walk, oxygen uptake (Figure 1) was similar for both men and women (t_(23)_=-1.34, *P *= 0.19). MET of the 1-mile walk was of moderate intensity using M1 and similar for men (range: 3.9-8.0 METs) and women (range: 4.3-7.1 METs; t_(23)_=-1.91, *P *= 0.07) (Figure 2c). M1 was lower than the MET for M2, which indicated vigorous intensity (t_(24)_=16.00, *P*<0.001) (Figure 2c), however, values were similar for men (range: 4.8-10.3 METs) and women (range: 5.7-9.1 METs; Figure 2c). All participants had METs higher than three during the one-mile walk using both M1 and M2 (Figure 3). 29% of the participants had METs higher than six (i.e. vigorous intensity exercise) using M1 and 64% using M2. The intensity of the walk, relevant to predicted maximal METs was similar for M1 and M2 at 62% (SD 13). However, men completed the walk at a lower intensity of maximum METs than women (men: 56% [SD 11]; women: 71% [SD 9]; t_(23)_=3.57, *P *= 0.002).

For the 1-mile walk using M1, 81.5 (SD 15.8) MET·mins^-1^ were completed, with no differences between men (83.0 [SD13.1] MET·min^-1^) and women (77.7 [SD 11.7] MET·min^-1^; t_(23)_=-0.12, *P*=0.31). The minimum MET minutes completed was 57.2 MET·min^-1^, with a maximum of 104.0 MET·min^-1^ per 1-mile walk using M1. M2 had a significantly higher value of 105.6 MET·min^-1^ for the 1-mile walk (t_(24)_=22.48, *P *< 0.001), with no differences between men (105.9 [SD 15.8] MET·min^-1^) and women (105.1 (SD 14.2) MET·mins^-1^; t_(23)_=-1.03, *P *= 0.31). Correlations demonstrated a significant moderate negative correlation between time to complete 1-mile and walking METs for both M1 (r=-0.66, *P *< 0.001) and M2 (r = -0.68, *P *< 0.001).

**Figure 4:**
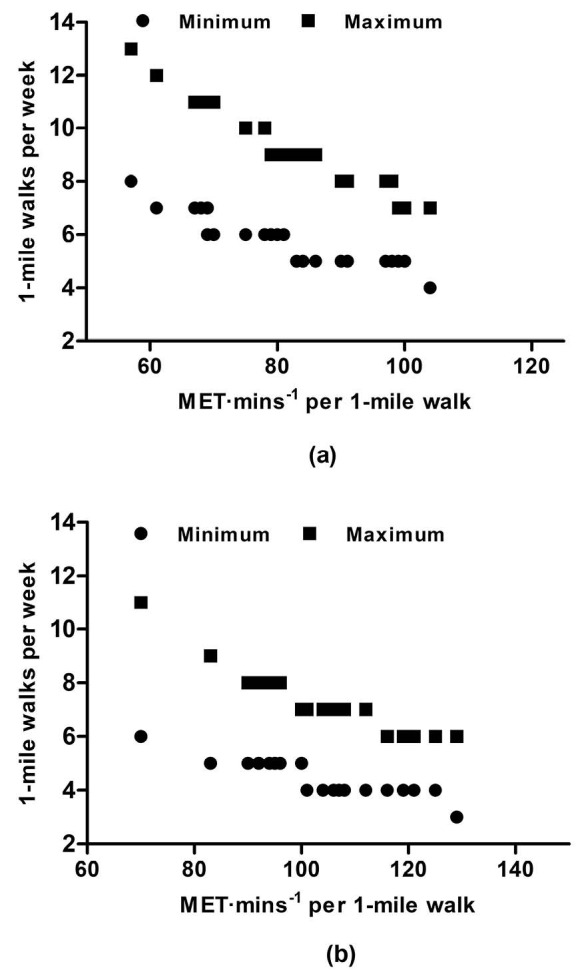
Relationship between MET·min^-1^ per 1-mile walk and the number of 1-mile walks per week to meet the minimum (450 MET·min^-1^·wk^-1^) and upper target (740 MET·min^-1^·wk^-1^) using a) Jetté et al^7^ (M1) and b) Byrne et al^8^ 1-MET values (M2).

Using M1, to obtain the minimum required 450 MET·min^-1^·wk^-1^ participants should complete a minimum of six 1-mile walks per week. To obtain the upper target of 750 MET·min^-1^·wk^-1^, participants should complete nine 1-mile walks per week, with no differences between men and women (lower target: t_(23)_=0.98, *P *= 0.33; upper target: t_(23)_= 0.77, *P *= 0.50) (Figure 4). When M2 is used for recommendations, the number of 1-mile walks required to meet both the lower and upper targets is significantly less (lower target: t_(24)_=-14.91, *P *< 0.001; upper target: t_(24)_= -15.56, *P *< 0.001), with no differences between men and women (lower target: t_(23)_=0.00, *P *= 1.00; upper target: t_(23)_= 0.72, *P *= 0.91). To acquire between 450 and 750 MET·min^-1^·wk^-1^using M2, participants should complete between four and seven 1-mile walks per week (Figure 4).

## Discussion

Findings from the present study extend and simplify the existing knowledge on exercise prescription and public health, indicating that instructions to walk 1-mile as fast as possible allow older adults to achieve a moderate to vigorous intensity. This observation enables us to recommend more explicit and manageable weekly walking exercise to promote and maintain health in older adults, with the completion of between four and nine 1-mile walks per week at a fast, constant pace.

The 1-mile walk is a distance used to estimate aerobic capacity.^14^ Walking is also an exercise modality promoted by many public health campaigns, for example Change for Life and Walking for Health in the United Kingdom, suggesting that achieving the recommendations reduces the risk of chronic diseases and maintains quality of life.^1,2^ Older adults in the present study completed the 1-mile walk in 14 minutes and 48 seconds (1.86 m·s^-1^/4.2 miles·h^-1^) when provided instructions similar to those for the Rockport 1-mile walk test.^9^ This was faster than participants of similar age in Bazzano et al^14^ (15 minutes 18 seconds; 1.74 m·s^-1^/ 3.9 m·h^-1^).Although the participant characteristics were similar between studies, V̇O_2_max in the present study (men: 36.6 mL∙kg^-1^∙min^-1^; women: 25.4 mL∙kg^-1^∙min^-1^) was greater than those in Bazzano et al^14^ (men: 28.0 mL∙kg^-1^∙min^-1^; women: 23.7 mL∙kg^-1^∙min^-1^) indicating a greater level of aerobic fitness and ability to complete 1-mile at a faster pace. The 1-mile walking speeds were similar to the maximal walking speeds of older adults in Bohannon,^15^ between a fast and maximal walking speed of those older adults in Fitzsimons et al^4^ and greater than the walking speeds used in Hall et al^9^ to assess the energy costs of walking in older adults.

The step frequency used to maintain these speeds were different between men (127 [SD 9] steps·min^-1^) and women (135 [SD 6] steps·min^-1^). This could be attributed to the fact that men were taller than women, have a longer stride length and therefore fewer steps to cover 1-mile.^16^ General recommendations suggest that a generic stride rate of 103 steps per minute equates to an intensity of 3-METs,^16,17^ a much lower stride rate than both men and women in the present study, and indicating an intensity higher than 3 METs. Using M1, participants were walking at 6 METs using a step rate of 130 per minute and 7 METs for the M2 method. Rowe et al^17^ suggest 4 METs and 5 METs require 120 steps per minute and 140 steps per minute, respectively, and for every 1 MET increase there is an increase in stride rate of 16-24 steps per minute. If these findings were applied to the participants in the present study, to achieve the 6-7 METs they would have a stride rate of 156-164 or 172-188 steps per minute, respectively.Such a step-rate would be difficult to achieve for the general population, and only a few participants were able to achieve a vigorous intensity walk within the study by Rowe et al.^17^ Utilising a step frequency of 130 steps per minute achieved a higher MET value for older adults in the present study (M2 7-METs for 1-mile), 40 steps per minute less than the suggestion by Rowe et al.^17^ One reason for this was that Rowe et al^17^ used the same method as M1 to calculate 1-MET. This was much higher than M2, and when divided into oxygen uptake, MET values were higher indicating the walk was more intense. Further to this, the MET values for both M1 and M2 could be higher than that calculated by Rowe et al^17^ because measured V̇O_2_ values during the walk were higher in the present study (19.4 mL·kg^-1^·min^-1^vs Rowe et al^17^: 14.7 mL·kg^-1^·min^-1^). This could be the result of differences in walk duration, 6-minutes as opposed to 12 to 19-minutes. These findings could be utilised by health and exercise practitioners when prescribing exercise intensity, providing older adults with clear instructions to maintain a step-rate of 127-135 steps per minute when walking, to elicit an appropriate intensity, facilitating meeting physical activity recommendations.

Heart rate during the walk also indicated a moderate-vigorous intensity (men: 116 beats∙min^-1^, 72% maxHR; women:134 beats∙min^-1^, 83% maxHR). Men completed the walk quicker than women, with a lower heart rate and percentage of their maximum, but this was reflected in their higher levels of physical fitness (36.6 vs. 25.4 mL∙kg^-1^∙min^-1^). This is similar to the results obtained by Bazzano et al,^14^ were men had a lower heart rate than women during the 1-mile walk. However, relative V̇O_2_ during the walk for both men and women was a similar intensity, suggesting a similar overall effort around 70%-75% of their V̇O_2_max.^14^

As mentioned before, 1-MET differed between M1 and M2 and caused the maximal METs and 1-mile walking METs to be greater for M2. These methods of predicting 1-MET were used, as opposed to a direct measurement, because they can be easily employed in the public domain by practitioners. Many participants exercised at a vigorous intensity during the 1-mile walk when M2 was used (64%), with 29% achieving vigorous intensity exercise when M1 was used. However, all individuals were 4 METs and above for both methods. This indicates that instructions to walk at a fast pace for 1-mile will at least achieve a moderate intensity, and facilitate the instructions given to older adults by exercise professionals to ensure sufficient physical activity participation and resultant physiological adaptations.^6^

The difference between M1 and M2 resting MET values suggest that the walk is more intense using M2, therefore, if they complete the walk in the same time, the MET·min^-1^ per walk is higher for M2. And, to achieve the recommended 450-750 MET∙min∙wk^-1^,M1 requires the accumulation of more 1-mile walks per week (6-9 vs. 4-7).

When comparing the proposed walking protocol to that completed by younger adults in Murphy et al,^18^ they walked at a speed of 1.64 (SD 0.71) m·s^-1^ with an oxygen uptake of 59% (SD 1.2) of V̇O_2_max and an average heart rate of 119 (SD 4) beats∙min^-1^ for 10-minutes. The walking speed and heart rate for the 1-mile bouts was greater for the older adults in the present study, i.e. 1.86 m·s^-1^ and 123 (SD 16) beats∙min^-1^ respectively. However, older adults in the present study exercised at a similar percentage of their V̇O_2_max (62 (SD 12.8) %) to those in Murphy et al,^18^ which concluded that brisk walking undertaken in accumulated sessions throughout the day can reduce post-prandial plasma triacylglycerol concentrations and increase fat oxidation. A later intervention study by this group used a similar protocol over the duration of 6-weeks, 10-minute bouts of brisk walking, 3-times per day, 5 d·wk^-1^ at 70%-80% predicted maximal heart rate.^3^This intervention improved predicted V̇O_2_max, sum of skin folds, waist, hip circumference and blood lipid profiles of middle-aged men and women (44.5 [SD 6.1] years).^3^ The overall concept is similar to the suggestions made in the present study, and therefore an intervention of similar duration could elicit similar results. However, participants in Murphy et al^3^ completed the recommended 150 min·wk^-1^ of moderate intensity exercise. Using M1 as a recommendation tool for older adults, they would have to complete between 90 and 133 min·wk^-1^ of exercise and 60-103 min·wk^-1^ for M2. This is less than those in Murphy et al,^3^ however it should be noted that a majority of individuals demonstrate vigorous intensity activity with M2. Recommendations suggest older adults can complete a minimum of 75 min·wk^-1^ vigorous activity per week, therefore a minimum of five 1-mile walks per week would reach this recommendation.^1^

Sedentary, overweight men and women (aged 40-65 years) benefitted from walking 12-miles, in approximately 3-sessions per week at 40%-55% or 65%-80% of V̇O_2_max.^19^ The later intensity prescription is similar to the current recommendations from M2, vigorous intensity, however more miles in fewer repetitions (3 per week). Slentz et al^19^ suggested that the number of calories expended is an important part of exercise prescription.Time required to expend calories depends on the fitness levels, with the lower fit individuals requiring more time and higher fit individuals requiring less time to expend a given number of calories. With our results, we observed a negative correlation between time to complete 1-mile and walking METs, indicating those that walk the 1-mile quicker will have a higher walking intensity. When aligned to the suggestion by Slentz et al,^19^ those more fit individuals are able to walk faster at a higher intensity, therefore require less walks, which is also reflective of the differences between M1 and M2 for calculating 1-MET. Public health exercise professionals should therefore promote the accumulation of more 1-mile walks to those older adults with a lower aerobic capacity to ensure they achieve the threshold for improving health.

## Conclusion

Many different parameters, i.e. frequency, intensity, duration and type of exercise, have to be considered in exercise guidelines for older adults, with some difficult to measure in the general public. Our observations suggest that if exercise/health professionals provide older adults instructions to walk 1-mile as fast as possible, they can achieve an intensity that is in line with recommendations for % HRmax, V̇O_2_max, and step frequency. In accordance to M2, older adults walk at a vigorous intensity for METs and exercise professionals working within public health, should encourage them to complete between four and seven 1-mile walks per week. M1 however requires moderate intensity walks of between 6 and 9, therefore when prescribing exercise to individuals with a lower aerobic capacity, who are unable to maintain a higher intensity for 1-mile, they should be encouraged to accumulate more 1-mile walks. Both methods do not directly measure 1-MET for individuals, but rather predict this, which is practical for use by the general public. Further research should directly measure 1-MET to ensure the predicted MET intensity is transferrable, in addition to determining the application of this across various settings. This should lead to future prospective studies in larger samples, encouraging older adults to progressively accumulate between seven and nine 1-mile walks per week.

## Ethical approval

Ethical approval for this study was provided by the University of Chichester Research Ethics Committee prior to its initialisation.

## Competing interests

There are no funding conflicts or other conflicts of interest to declare.

## Authors’ contributions

MLG and METW proposed the study. Data collection and analysis was performed by MLG, with manuscript drafting and data interpretations by both authors.

## Acknowledgments

The authors wish to thank all the participants for their involvement in the study.
